# Role of Epigallocatechin Gallate in Glucose, Lipid, and Protein Metabolism and L-Theanine in the Metabolism-Regulatory Effects of Epigallocatechin Gallate

**DOI:** 10.3390/nu13114120

**Published:** 2021-11-17

**Authors:** Ling Lin, Li Zeng, An Liu, Dongyin Yuan, Yingqi Peng, Sheng Zhang, Yinhua Li, Jinhua Chen, Wenjun Xiao, Zhihua Gong

**Affiliations:** 1 Key Laboratory of Tea Science of Ministry of Education, Hunan Agricultural University, Changsha 410128, China; linling1371752238@163.com (L.L.); Liuan-62@foxmail.com (A.L.); ydy19941005@163.com (D.Y.); pengyingqi2021@163.com (Y.P.); zhangsheng@hunau.edu.cn (S.Z.); liyinhua1012@hunau.edu.cn (Y.L.); hnjhchen@21cn.com (J.C.); gzh041211@163.com (Z.G.); 2 National Research Center of Engineering Technology for Utilization of Botanical Functional Ingredients, Hunan Agricultural University, Changsha 410128, China; 3 Co-Innovation Center of Education Ministry for Utilization of Botanical Functional Ingredients, Hunan Agricultural University, Changsha 410128, China; 4 School of Pharmacy, Shaoyang University, Shaoyang 422002, China; zanan1230@163.com

**Keywords:** epigallocatechin gallate, L-theanine, AMP-activated protein kinase pathway, insulin pathway, nutrient metabolism

## Abstract

Epigallocatechin gallate (EGCG) and L-theanine (LTA) are important bioactive components in tea that have shown promising effects on nutrient metabolism. However, whether EGCG alone or combined with LTA can regulate the glucose, lipid, and protein metabolism of healthy rats remains unclear. Therefore, we treated healthy rats with EGCG or the combination of EGCG and LTA (EGCG+LTA) to investigate the effects of EGCG on nutrient metabolism and the role of LTA in the metabolism-regulatory effects of EGCG. The results showed that compared with the control group, EGCG activated insulin and AMP-activated protein kinase (AMPK) signals, thus regulating glucose, lipid, and protein metabolism. Compared with EGCG, EGCG+LTA enhanced hepatic and muscle glycogen levels and suppressed phosphorylation of AMPK, glycogen synthase 2, mammalian target of rapamycin, and ribosomal protein S6 kinase. In addition, EGCG+LTA inhibited the expression of liver kinase B1, insulin receptor and insulin receptor substrate, and promoted the phosphorylation level of acetyl-CoA carboxylase. Furthermore, both EGCG and EGCG+LTA were harmless for young rats. In conclusion, EGCG activated AMPK and insulin pathways, thereby promoting glycolysis, glycogen, and protein synthesis and inhibiting fatty acid (FA) and cholesterol synthesis. However, LTA cooperated with EGCG to promote glycogen metabolism and suppressed the effect EGCG on FA and protein synthesis via AMPK signals.

## 1. Introduction

Glucose, lipid, and protein metabolism is essential for growth. It is a complex process requiring tight coordination of various signals, and it can be regulated by exogenous additives.

L-theanine (LTA), a non-protein amino acid in tea, has shown antidepressant, immunoregulation, learning and cognition improvement, and other beneficial activities [1,2,3,4,5]. Recently, studies focusing on LTA have revealed its ability to regulate nutrient metabolism. It has been reported that LTA affects the absorption of glucose, lipids, and amino acids by regulating the expression of intestinal glucose, fatty acid (FA), and amino-acid transporters [6]. Zheng et al. [7] showed that LTA can decrease the concentrations of triglycerides (TGs) and non-esterified fatty acids in mouse sera. We previously showed that LTA improves the absorption and utilization efficiency of amino acids in normal mice [8] and regulates the metabolism of short-chain FAs [2]. We also showed that LTA regulates glucose, lipid, and protein metabolism via the AMP-activated protein kinase (AMPK) and insulin pathway [9].

Epigallocatechin gallate (EGCG), an important component of green tea, is a nutraceutical with strong antioxidant, anticarcinogenic, anti-inflammatory, and cardioprotective bioactivities [10,11,12,13]. Importantly, EGCG has promising effects on nutrient metabolism [14]. The consumption of green tea extract or EGCG significantly reduces gain of body weight and adipose tissues, decreases blood glucose or insulin levels, and increases insulin sensitivity or glucose tolerance [15]. The effects of EGCG on metabolic syndrome or obesity can be achieved via both the AMPK and insulin pathways. Furthermore, it has been reported that EGCG can promote glycogen synthesis, phosphorylation of glycogen synthase kinase (GSK) 3β and glycogen synthase (GYS), and inhibit lipogenesis by promoting the phosphorylation of AMPK and acetyl-CoA carboxylase (ACC) [16]. Li et al. [15] found that 50 and 100 mg/kg EGCG can reduce obesity and epididymal white adipose tissue weight partially by activating AMPK in mice. Moreover, the effects of EGCG on AMPK activation are realized by activating liver kinase B1 (LKB1) [17]. In addition, EGCG can regulate nutrient metabolism via insulin signals. Li et al. [18] showed that EGCG ameliorates free fatty acid (FFA)-induced peripheral insulin resistance through activating the insulin signaling and AMPK pathways. However, previous studies of EGCG on nutrient metabolism have mostly focused on sub-healthy groups; yet, maintaining a normal metabolism of a healthy body in the modern dietary structure is also critical for life activities, and the regulatory effects of EGCG on a healthy body are rare.

Recently, the synergistic biological activity of different tea compounds has attracted much attention. A previous study revealed synergistic effects of EGCG, LTA, and caffeine on sperm viability [19]. Yang et al. [20] revealed that co-administration of EGCG and caffeine can ameliorate obesity and non-alcoholic fatty liver disease in obese rat. More recently, we found that EGCG cooperated with LTA in promoting the absorption and utilization of amino acids [8], which indicates that co-administration with other tea components may affect their functions. Based on these findings, we speculated that co-administered LTA affects the EGCG-mediated regulation of glucose, lipid, and protein metabolism.

Therefore, in this study, we treated healthy rats with EGCG and the combination of EGCG and LTA (EGCG+LTA), respectively, to investigate the effects and molecular mechanisms of EGCG on nutrients metabolism and, more importantly, the role of LTA on the regulatory effects of EGCG on glucose, lipid, and protein metabolism.

## 2. Materials and Methods

### 2.1. Materials and Reagents

EGCG and LTA (both with over 98% purity) were purchased from Hunan Sunfull Bio-Tech Co., Ltd. (Changsha, China). Ethanol absolute, neutral balsam, xylene, hydrochloric acid, and isopropanol were purchased from Sinopharm Chemical Reagent Co., Ltd. (Shanghai, China). Tris, sodium dodecyl sulfate, acrylamide, Tween 20, protease inhibitor, and protein phosphatase inhibitor were purchased from Sigma–Aldrich (St. Louis, MO, USA). Secondary antibody for horseradish peroxidase (HRP, goat anti-rabbit) and primary antibodies against β-actin, GYS2, p-GSK-3β, GSK-3β, 3-hydroxy-3-methylglutaryl-CoA reductase (HMGCR), ACC1, FA synthase (FAS), ribosomal protein S6 kinase (p70S6K), ribosomal protein S6 (S6), mammalian rapamycin (mTOR), LKB1, phosphatidylinositol-4,5-bisphosphate3-kinase (PI3K), activated protein kinase B (AKT), and insulin receptor (INSR) were purchased from Proteintech (Rosemont, IL, USA). Primary antibodies against p-AMPK, p-AKT, p-ACC1, p-mTOR, p-p70S6K, p-S6, insulin receptor substrate (IRS), AMPK, and phosphofructokinase liver type (PFKL) were purchased from Abcam (Cambridge, UK).

### 2.2. Animals

Twenty-four Sprague–Dawley (*Rattus norvegicus*) male rats (specific pathogen-free grade, 4 weeks old) were purchased from Hunan SJA Laboratory Animal Co., Ltd. (Changsha, China). All animals and experimental procedures were performed according to the National Institutes of Health Guidelines on the Use of Laboratory Animals (NIH Publication No. 85–23 Rev. 1985). All protocols were approved by the Ethics Committee of Hunan Agriculture University (registration number: 015063506, Changsha, China).

### 2.3. Experimental Design

All rats were housed in a temperature-controlled (25 ± 2 °C) room with relatively constant humidity (50–70%) and a 12 h light/dark cycle. After a seven-day adaption with free access to food and water, rats were randomly divided into three groups (*n* = 8): the control, EGCG treatment, and EGCG+LTA treatment groups. Rats in the EGCG treatment groups were administered 80 mg/kg of EGCG by gastric irrigation. Then, according to the proportion of EGCG and LTA in green tea powder (EGCG: LTA = 4:1), rats in the EGCG+LTA group were intragastrically administered a mixture of 80 mg/kg of EGCG and 20 mg/kg of LTA; rats in the control group were administered physiological saline. The administration volume was adjusted to 1 mL/100 g body weight, and the treatment lasted for 28 days. On day 29, all rats were denied feed overnight and were anesthetized with sodium pentobarbital. Blood samples were collected from the abdominal aorta, allowed to stand for 30 min, and then centrifuged at 3500 rpm for 10 min (4 °C). Serum samples were collected and stored at −80 °C. Rat livers and skeletal muscles were harvested, weighed, and stored at −80 °C until use.

### 2.4. Liver Histological Analysis

Immediately after weighing the livers, the same position on the left lobes were sectioned (approximately 2–3 cm) and fixed in 4% paraformaldehyde for more than 24 h. Fixed livers were dehydrated using gradient alcohol, embedded with paraffin, sliced into 4–5 μm sections, and stained with hematoxylin and eosin (HE). The histological sections were analyzed using light microscopy (200× magnification).

### 2.5. Serum Biochemical Indexes and Enzyme Activity Measurement

The contents of serum total protein (TP), albumin (Alb), triglyceride (TG), total cholesterol (TC), low-density lipoprotein cholesterol (LDL-C), high-density lipoprotein cholesterol (HDL-C), and glucose as well as serum alanine aminotransferase (ALT) and aspartate transaminase (AST) activities were measured using a Varioskan Flash full-wavelength scanning multifunctional reader (Thermo Fisher Scientific Inc., Waltham, MA, USA) as described by the manufacturer (Nanjing Jiancheng Bioengineering Institute, Nanjing, China). Rat livers and skeletal muscles were powdered in liquid nitrogen and reacted with extracts to determine phosphoenolpyruvate carboxykinase (PEPCK), citrate synthase (CS), PFK, glycogen phosphorylase a (GPa), ACC, FAS, carnitine palmitoyl transferase-1 (CPT-1), and glycogen contents using the respective assay kits as described by the manufacturer (Suzhou Comin Biotechnology Co., Ltd., Suzhou, China).

An ELISA test was performed to determine the contents of insulin-like growth factor 1 (IGF-1) and insulin in serum according to the manufacturer’s instructions (Huamei Biotechnology Co., Ltd., Wuhan, China).

### 2.6. Western Blotting

Western blotting was performed as described in a previous report [21]. In brief, proteins were extracted and separated using gel electrophoresis and subsequently transferred to nitrocellulose membranes. Next, the membranes were incubated with primary and secondary antibodies, and the results were analyzed using the Quantity One software (Version 4.6.6; Bio-Rad, Hercules, CA, USA).

### 2.7. Statistical Analysis

Data analysis was performed using SPSS software (version 24.0; IBM, Armonk, NY, USA), and all graphs were plotted by GraphPad Prism (version 8.0.1; GraphPad Software, San Diego, CA, USA). Results are expressed as the mean ± SE. Differences among more than two groups were determined by one-way analysis of variance (ANOVA), and multiple comparisons were performed by LSD. Values with *p* < 0.05 were considered statistically significant.

## 3. Results

### 3.1. Neither EGCG Nor EGCG+LTA Was Toxic to Rats

**Figure 1:**
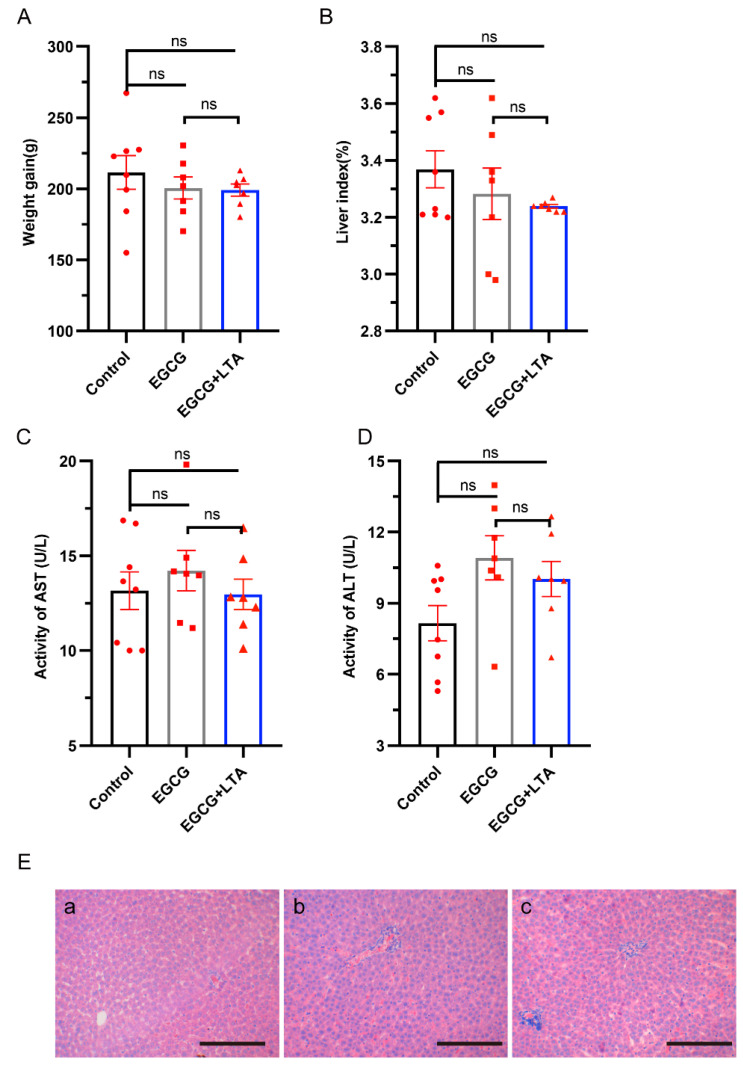
Neither EGCG nor the combination of EGCG and LTA (EGCG+LTA) was toxic to Rats. (**A**) Average weight gain of rats over 28 days. (**B**) Liver index of rats in different groups. Activities of serum AST (**C**) and ALT (**D**) among the three groups. (**E**) Hematoxylin and eosin staining of liver tissue sections (scale bar: 100 μm): (**a**) control group, (**b**) EGCG treatment, and (**c**) EGCG+LTA treatment group. Values are expressed as the mean ± SE. ns: no significant difference.

After a 28 day treatment with different bioactive ingredients from tea, rats exhibited body weight gain, but no significant differences were observed among different groups (*p* > 0.05) (Figure 1A). In addition, neither EGCG nor EGCG+LTA altered the liver index (the ratio of liver weight to body weight) (Figure 1B). We further investigated the effects of EGCG and EGCG+LTA on young rats by staining liver sections with HE. We found that in the EGCG treated group, the nuclei were more prominent than those in the control and EGCG+LTA treated groups (Figure 1F). The activities of serum AST and ALT were slightly but not significantly elevated by 80 mg/kg EGCG (*p* > 0.05; Figure 1C,D).

### 3.2. LTA Cooperated with EGCG to Promote Glycogen Synthesis

**Figure 2:**
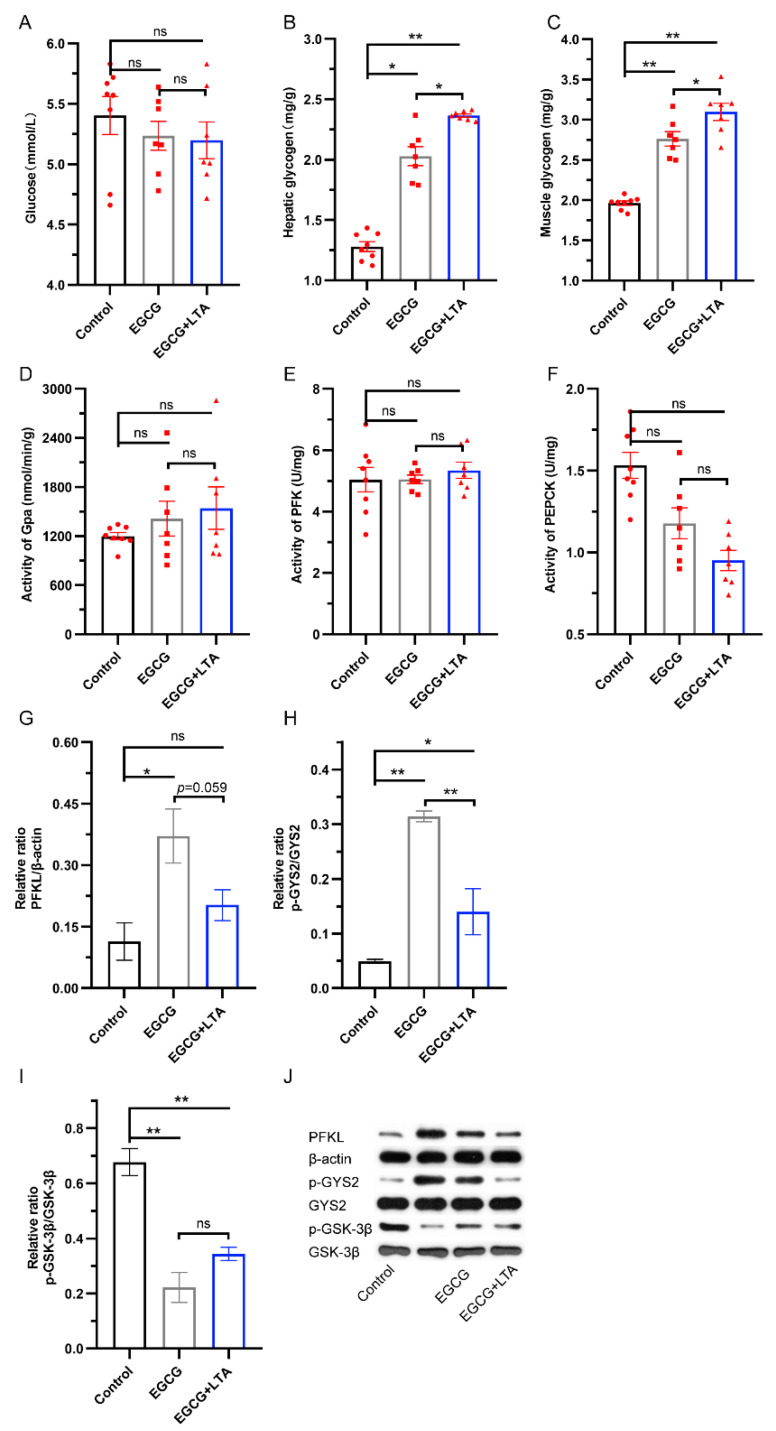
LTA cooperated with EGCG to promote glycogen synthesis. Blood glucose (**A**), hepatic glycogen (**B**), and muscle glycogen (**C**) contents in different groups. Activities of liver GPa (**D**), PFK (**E**), and PEPCK (**F**) among the three groups. Relative protein expression of PFKL (**G**) and phosphorylation level of GYS2 (**H**) and GSK-3β (**I**) in rat livers (**J**). Values are expressed as the mean ± SE. * *p* < 0.05 and ** *p* < 0.01. ns: no significant difference.

Figure 2 shows that EGCG and EGCG+LTA did not significantly affect blood glucose content or GPa, PFK, and PEPCK activities (Figure 2A,D–F). Compared with those in the control group, the contents of hepatic and muscle glycogen were increased by EGCG and EGCG+LTA treatments, and the effects of EGCG+LTA on glycogen content were more profound than those of EGCG alone (*p <* 0.05; Figure 2B,C), indicating that both EGCG and LTA positively affected glycogen synthesis. We analyzed the proteins related to glycolysis and glycogen synthesis. As shown in Figure 2, compared with the control group, 80 mg/kg EGCG promoted the expression of PFKL, and the co-administration of ECGC with 20 mg/kg LTA did not alter the effect of EGCG on PFKL expression (Figure 2G,J). Phosphorylation of GSK-3β was downregulated in the EGCG and EGCG+LTA groups compared to that in the control group (Figure 2I,J). Conversely, both EGCG and EGCG+LTA enhanced GYS2 phosphorylation. When compared to EGCG treatment, EGCG and LTA co-administration decreased GYS2 phosphorylation (Figure 2H,J) and elevated glycogen content (Figure 2B,C).

### 3.3. EGCG and LTA Co-Administration Affected Lipid Synthesis Less Significantly Than EGCG Alone

**Figure 3:**
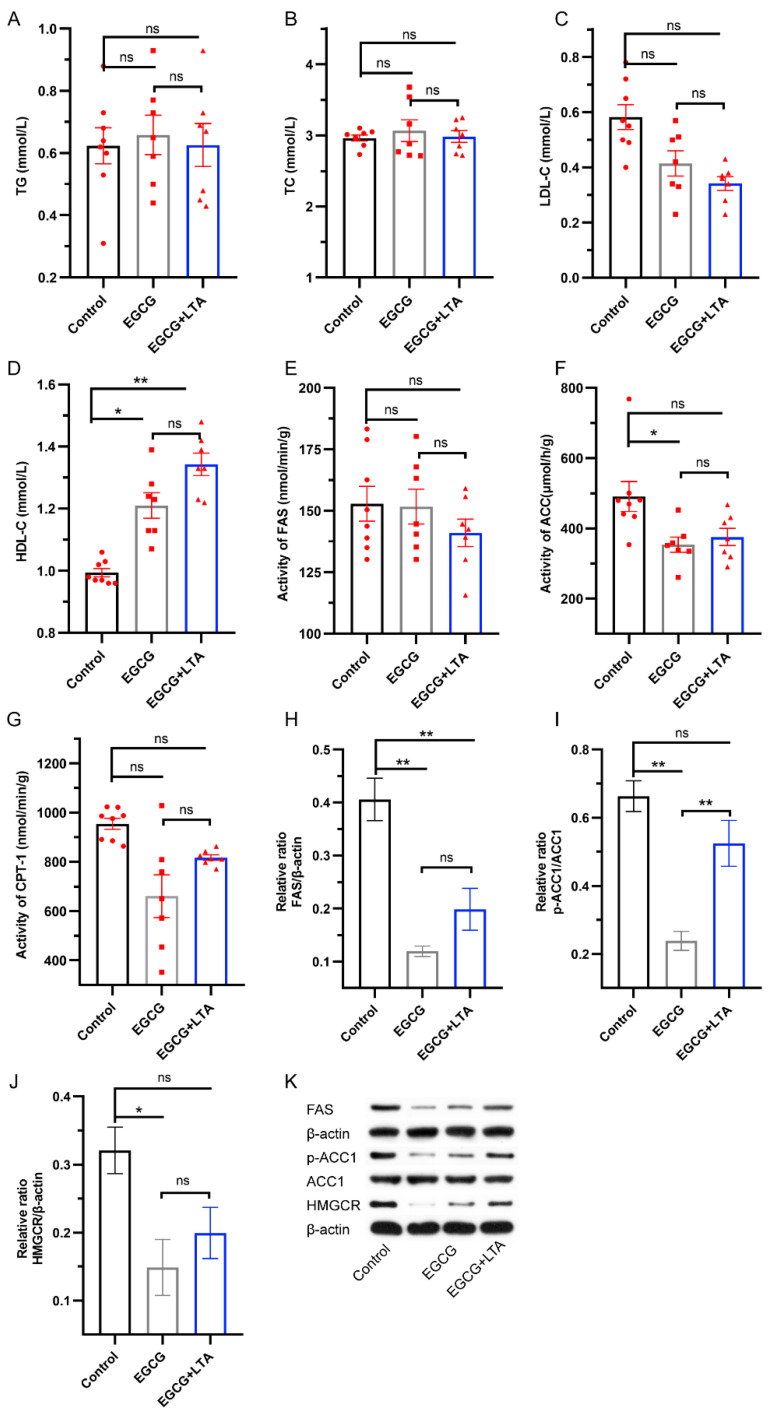
EGCG and LTA co-administration affected lipid synthesis less significantly than EGCG alone. Serum TG (**A**), TC (**B**), LDL-C (**C**), and HDL-C (**D**) contents in different groups. The effects of different treatments on the activities of hepatic FAS (**E**), ACC (**F**), and CPT-1 (**G**). Effects of different treatments on protein expression of FAS (**H**), p-ACC1 (**I**), and HMGCR (**J**) in rat livers (**K**). Values are expressed as the mean ± SE. * *p* < 0.05 and ** *p* < 0.01. ns: no significant difference.

The effects of EGCG and EGCG+LTA on lipid metabolism are depicted in Figure 3. TG, TC, and LDL-C contents were unaltered by EGCG and EGCG+LTA (Figure 3A–C). Compared to that in the control group, HDL-C content was enhanced by EGCG and EGCG+LTA, whereas no significant effect was observed between the EGCG and EGCG+LTA groups (Figure 3D). We then analyzed the activities of key enzymes involved in lipogenesis and lipolysis. As shown in Figure 3, the activities of FAS and CPT-1 remained relatively constant, irrespective of the treatment implemented (Figure 3E,G). However, ACC, a key rate-limiting enzyme in lipogenesis, was inhibited by EGCG rather than EGCG+LTA (Figure 3F). Additionally, compared to that in the control group, the expression of FAS was significantly downregulated by EGCG and EGCG+LTA treatment (Figure 3H). Moreover, EGCG treatment downregulated the expression of p-ACC1 and HMGCR when compared with that in the control group (Figure 3I,K). However, compared with that in the EGCG group, the expression of these three proteins was slightly elevated by EGCG+LTA, with the expression of p-ACC1 being significantly different (*p* < 0.01).

### 3.4. Protein Synthesis Promoted by EGCG Was Weakened by LTA

**Figure 4:**
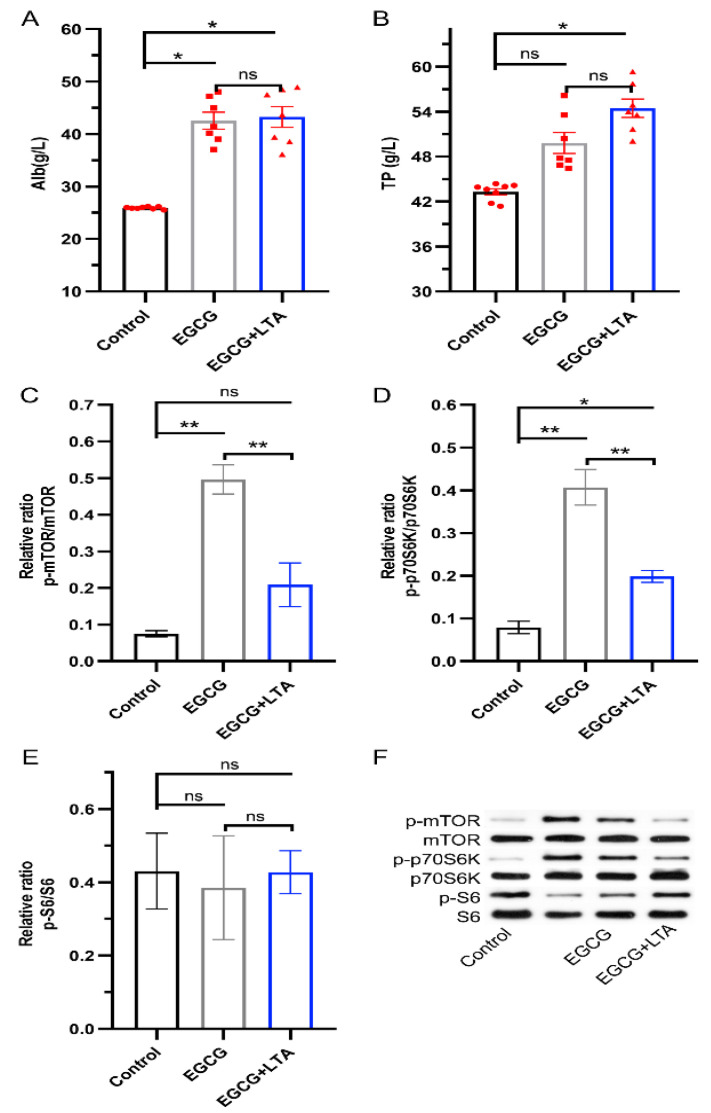
Protein synthesis promoted by EGCG was weakened by LTA. Serum Alb (**A**) and TP (**B**) contents in different groups. Phosphorylation levels of mTOR (**C**), p70S6K (**D**), and S6 (**E**) in rat livers (**F**). Data are expressed as the mean ± SE. * *p* < 0.05 and ** *p* < 0.01. ns: no significant difference.

As depicted in Figure 4, the EGCG and EGCG+LTA groups had a higher serum Alb content (*p* < 0.05) than the control group, but no significant difference was observed between the EGCG and EGCG+LTA groups (Figure 4A). Further, TP content in the EGCG+LTA group was higher than that in the control group, whereas it remained unaltered in the EGCG group when compared with that in the control group (Figure 4B). Additionally, compared to that in the control group, the phosphorylation level of mTOR was enhanced by EGCG (Figure 4C,F), and the phosphorylation of its downstream protein, p70S6K, was promoted by both EGCG and EGCG+LTA (Figure 4D,F). In addition, co-administration of EGCG and LTA decreased the expression level of p-mTOR and p-p70S6K when compared with EGCG treatment.

### 3.5. AMPK Signals Played an Important Role in the Metabolism-Regulatory Effects of EGCG and EGCG+LTA

**Figure 5:**
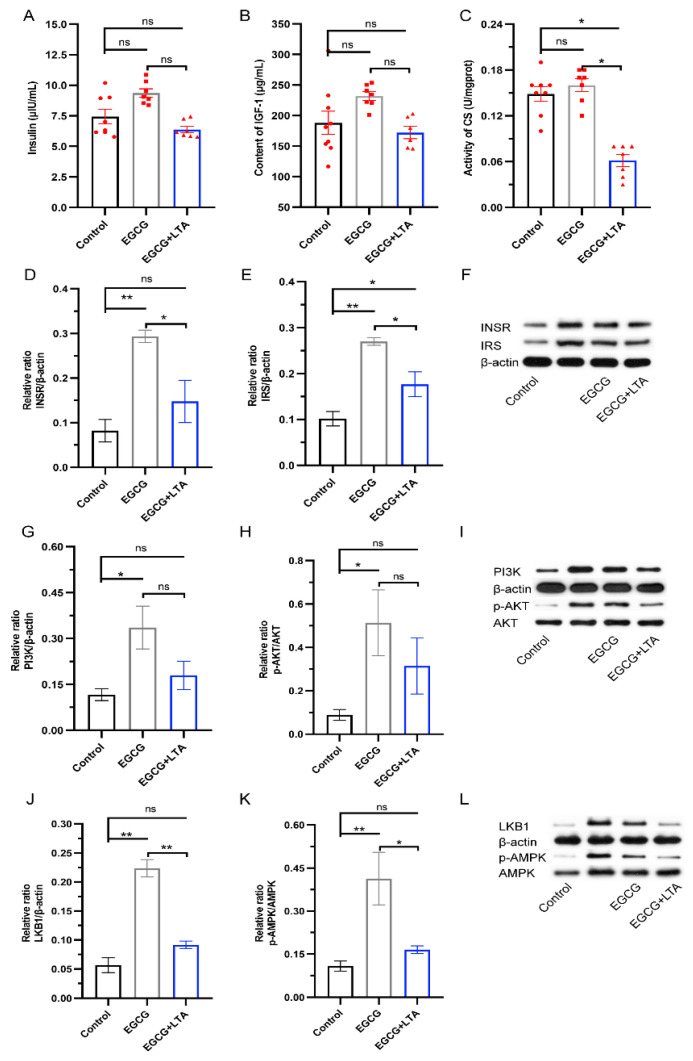
AMPK signals played an important role in the metabolism-regulatory effects of EGCG and EGCG+LTA. Serum insulin (**A**) and IGF-1 (**B**) contents and hepatic CS activity (**C**) in different groups. Relative protein expression of INSR (**D**) and IRS (**E**) in rat livers (**F**). Effects of different treatments on the expression of PI3K (**G**) and p-AKT (**H**) proteins (**I**). Relative expression levels of LKB1 (**J**) and p-AMPK (**K**) in rat livers (**L**). Values are expressed as the mean ± SE. * *p* < 0.05 and ** *p* < 0.01. ns: no significant difference.

To further investigate the mechanisms underlying the effects of EGCG and EGCG+LTA on glycogen, lipid, and protein metabolism, we examined indexes related to the insulin and AMPK pathways. Figure 5A,B show that EGCG and EGCG+LTA did not alter serum insulin and IGF-1 (*p* > 0.05). However, the activity of a key enzyme in the tricarboxylic acid cycle, CS, was decreased by EGCG+LTA when compared to that in both the control and EGCG groups (Figure 5C). Compared to that in the control group, the expression of INSR was enhanced by EGCG (Figure 5D), and the expression of its downstream protein, IRS, was promoted by both EGCG and EGCG+LTA (Figure 5E). Moreover, compared to EGCG alone, co-administration of EGCG and LTA decreased the expression of INSR and IRS (Figure 5D–F). The expression of PI3K and p-AKT was also enhanced by EGCG compared to that in the control group, and no statistical significance was observed between the EGCG and EGCG+LTA groups (Figure 5G–I). As depicted in Figure 5J–L, the expression of LKB1 and p-AMPK was significantly upregulated by EGCG rather than EGCG+LTA when compared with that in the control group. Additionally, the EGCG+LTA group showed lower LKB1 and p-AMPK expression levels than did the EGCG group (*p* < 0.05; Figure 5J–L).

## 4. Discussion

Growth is accompanied by weight gain, and this process requires large amounts of energy. Here, rats in different groups exhibited similar average weight gain. This finding differs from those of previous studies in which EGCG significantly suppressed weight gain of mice [22,23]. This might have been because we used young rats, whereas these studies used adult mice. The liver is an important organ involved in metabolism. A high dose of EGCG can trigger acute toxicity in rat liver cells and evoke hepatotoxicity, with increased levels of serum ALT and AST [22,24]. However, HE staining, liver indexes, and activities of serum AST and ALT in the present study showed that 80 mg/kg EGCG was safe, which is consistent with previous studies in which a low dose of EGCG induced no toxicity [25,26]. Moreover, co-administration of 80 mg/kg EGCG and 20 mg/kg LTA, the proportion present in green tea powder (EGCG: LTA = 4:1), was also harmless to young rats.

The regulation of AMPK and insulin signals is indispensable for glucose, lipid, and protein metabolism. EGCG has been reported to activate the AMPK signaling pathway by phosphorylating LKB1 [17], and this was verified in our study. In addition, ECGG promoted the expression of proteins in the insulin pathway, such as IRS and INSR, indicating that the insulin signal was also activated by EGCG and this has been observed in a previous study in which EGCG suppressed glucotoxicity by activating IRS2 and AMPK signals [27]. PI3K, an important kinase downstream of insulin signals and the activity of which is clearly necessary for insulin action [28], was unaltered by LTA. In addition, AKT phosphorylation did not significantly differ between the EGCG and EGCG+LTA groups, indicating that the influence of LTA on EGCG is not via the insulin signaling pathway, as the majority of the actions of insulin, especially those involving nutrient metabolism, were amplified by AKT [29]. Conversely, LTA attenuated the expression of both LKB1 and p-AMPK, and it has been reported that activated AMPK directly phosphorylates key factors, such as ACC, HMGCR, GYS, and mTOR, involved in multiple pathways to regulate energy balance [30], which suggests that the role of LTA in the effect of EGCG on nutrient metabolism may be achieved by AMPK signaling.

Activated AMPK and insulin regulate downstream signals to alter glucose, lipid, and protein metabolism, thus maintaining energy balance. Generally, activated AMPK increases glycolysis through two main pathways [31,32]. The first is achieved by activating PFK phosphorylating and activating PFK-2 [33], which is a rate controlling enzyme in glycolysis. Results in this study indicate that EGCG promotes glycolysis through the AMPK/PFK pathway. The second is achieved by phosphorylating and activating phosphorylase kinase, which then phosphorylates and activates Gpa, an enzyme that controls glycogenolysis and catalyzes the production of substrates for glycolysis [34,35]. Obviously, the activity of Gpa remained constant, regardless of the treatment implemented, indicating that EGCG merely increased glycolysis by PFK in this study. In addition, neither the expression of PFK nor the activity of Gpa was altered by co-administration with LTA, which is a little different from the observation in our previous study in which 100 mg/kg LTA promoted glycolysis via PFK [9]. This discrepancy might be due to the difference in doses between the two studies.

Glycogen synthesis requires catalysis by glycogen synthase, which is encoded by *GYS*, whereas GYS is phosphorylated and inactivated by GSK-3β [36]. Furthermore, GSK3 activity is controlled by insulin, which inactivates GSK3 and consequently promotes GYS phosphorylation [37]. Here, both EGCG and EGCG+LTA inhibited the phosphorylation of GSK-3β and elevated p-GYS2 expression. According to a previous study [38], this should result in a higher glycogen level in the EGCG and EGCG+LTA groups, and our results confirmed this. Apart from insulin signals, activated AMPK can phosphorylate and inhibit GYS, thus inhibiting glycogen synthesis [39]. Compared to those in the EGCG group, both p-AMPK and p-GYS2 were decreased by EGCG+LTA, and the content of hepatic and muscle glycogen was elevated, which suggests that AMPK signal was involved in the effect of LTA on EGCG regarding glycogen synthesis. That is, both EGCG and EGCG+LTA promoted glycogen synthesis in which EGCG promoted glycogen synthesis via the INSR/IRS/PI3K/AKT/GSK−3β/GYS pathway, while LTA cooperated with EGCG on glycogen synthesis through the AMPK/GYS signal.

FA synthesis is a highly regulated multistep process involving distinct sets of enzymatic reactions, of which FAS and ACC catalysis are two rate-limiting steps. Consistent with ACC activity, EGCG suppressed ACC1 phosphorylation and FAS expression compared with the control, indicating that EGCG inhibited FA synthesis via AMPK/ACC1 signaling, because activated AMPK directly phosphorylates and suppresses ACC1/2 [40]. This is also a key mechanism underlying the anti-obesity effect of EGCG [41,42]. Compared with EGCG, the expression level of p-ACC1 was elevated by EGCG+LTA; by contrast, the expression of p-AMPK was decreased by EGCG+LTA. This showed that LTA attenuates the effect of EGCG on FA synthesis via the AMPK/ACC1 pathway. Except for FA metabolism, lipid metabolism also involves the biosynthesis and degradation of cholesterol. HMGCR, a rate-limiting enzyme that catalyzes the de novo synthesis of cholesterol in vivo [43], was inhibited by EGCG, and AMPK stimulation leads to inhibition of HMGCR [44]. Videlicet, EGCG simultaneously suppressed FA and cholesterol synthesis through AMPK signaling, while co-administration with LTA weakened these effects of EGCG.

The mTOR pathway, a well-conserved pathway that regulates growth and autophagy, was confirmed to regulate protein synthesis [45]. As an important signal downstream of the insulin and AMPK pathways, mTOR accepts signals and responds rapidly by activating or inhibiting its downstream kinases [46,47]. More specifically, AKT, activated by insulin or IGF-1, activates mTORC1 and the downstream p70S6K, thus promoting protein synthesis [48]. Conversely, activated AMPK inhibits mTORC1 phosphorylation and suppresses protein synthesis [46,49]. Clearly, EGCG promoted mTOR and p70S6K phosphorylation. Serum Alb and TP contents were also simultaneously elevated; these are important parameters for evaluating animal health and are also used to evaluate the status of protein absorption and metabolism [50]. These results indicated that EGCG promotes protein synthesis via the INSR/IRS/PI3K/AKT/mTORC1 pathway. Conversely, the expression levels of p-mTOR and p-p70S6K was decreased by EGCG+LTA when compared with those in the EGCG group. This result, combined with the expression of p-AMPK, suggests that LTA suppressed protein synthesis through AMPK/mTOR signaling.

As indicated above, apart from its known effects on glucose and lipid metabolism [18,51], EGCG is also effective in protein metabolism in healthy rats. However, LTA attenuated the effect of EGCG on FA and protein synthesis through AMPK signals. This result differs from that of our previous study in which EGCG cooperated with LTA in promoting the absorption and use of amino acids [8]. The discrepancy might result from the transformation of glucose, lipid, and proteins, which plays an important role in maintaining energy balance; in addition, the previous study only investigated the metabolism of amino acids. However, the exact mechanisms underlying this discrepancy remain undetermined, and modern molecular biology can be used to investigate the pathways and mechanisms involved. Moreover, there are other ingredients in tea, such as caffeine, tea pigments, and tea polysaccharides, and whether a combination of these components or tea itself can regulate nutrient metabolism can be explored in future studies. 

## 5. Conclusions

**Figure 6:**
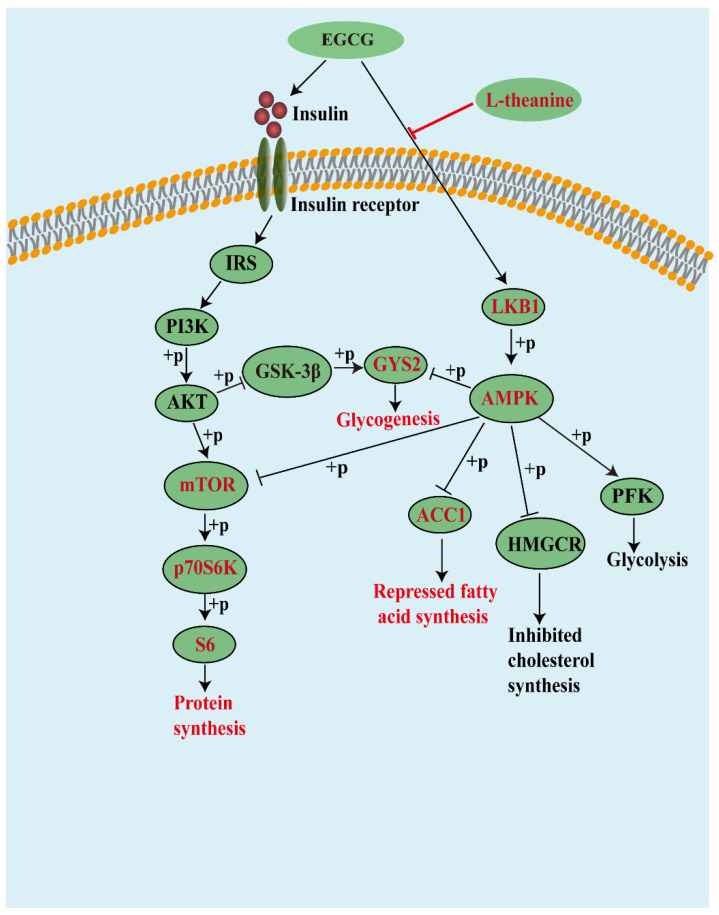
Schematic diagram of the mechanism.

EGCG promoted glycolysis via the LKB1/AMPK/PFK pathway, promoted glycogen synthesis through the INSR/IRS/PI3K/AKT/GSK−3β/GYS pathway, and inhibited FA and cholesterol synthesis via the LKB1/AMPK/ACC1 and LKB1/AMPK/HMGCR pathways. Moreover, it could promote protein synthesis through the INSR/IRS/PI3K/AKT/mTORC1 pathway. LTA cooperated with EGCG on glycogen synthesis via the AMPK/GYS signal, attenuated the effect of EGCG on FA and protein synthesis via the AMPK/ACC and AMPK/mTORC1 signals, respectively (Figure 6).

## Data Availability

Not applicable.
